# Secondary phosphorylation in myocytes expressing FLAG-tagged and non-tagged phospho-mimetic cardiac troponin I

**DOI:** 10.1016/j.dib.2017.09.066

**Published:** 2017-10-05

**Authors:** Sarah E. Lang, Tamara K. Stevenson, Tabea M. Schatz, Brandon J. Biesiadecki, Margaret V. Westfall

**Affiliations:** a Department of Cardiac Surgery, University of Michigan, Ann Arbor, MI 48109, USA; b Program in Cellular and Molecular Biology, University of Michigan, Ann Arbor, MI 48109, USA; c Department of Physiology and Cell Biology and Davis Heart and Lung Research Institute, The Ohio State University, Columbus, OH 43210, USA

## Abstract

Secondary phosphorylation develops in myocytes expressing phospho-mimetic cardiac troponin I (cTnI) but it is not known whether multiple substitutions (e.g. cTnISDTD and cTnIS4D) cause preferential phosphorylation of the remaining endogenous or the phospho-mimetic cTnI in intact myocytes. Western analysis was performed to determine whether the FLAG/total cTnI ratios are similar for phosphorylated versus total cTnI in myocytes expressing phospho-mimetic cTnI with Asp(D) substitutions at S43/45 plus S23/24 (cTnIS4D) or T144 (cTnISDTD). Representative Western analysis of phosphorylated S23/24 (p-S23/24) and S150 (p-S150) are presented along with re-probes using an antibody which detects all cTnI (MAB1691 Ab). The level of p-S150 also is compared to results obtained using single S43D and/or S45D phospho-mimetic substitutions. These results are discussed in more detail in Lang et al. [1]

**Specifications Table**

**Table:** 

Subject area	*Biochemistry*
More specific subject area	*Sarcomere signaling*
Type of data	*figures*
How data was acquired	*Western analysis using GelDoc MP imager and Quantity One software (BioRad).*
Data format	*Raw and analyzed*
Experimental factors	*Recombinant adenovirus was used for gene transfer of each cTnI substitution into primary cardiac myocytes isolated from rat hearts. These exogenous constructs replace endogenous cTnI in the sarcomere of myocytes over 4 days in culture.*
Experimental features	*Secondary phosphorylation of cTnI at S23/24 (p-S23/24) and S150 (p-S150) are analyzed by Western blot and compared in myocytes expressing cTnI with phospho-mimetic substitutions at one or more PKC-targeted sites. The*Fig. 1(panel A) shows p-S23/24 and p-S150 in myocytes expressing FLAG-tagged and non-tagged phospho-mimetic cTnI. Quantitative analysis of the %FLAG detected using the p-S23/24 versus cTnI antibodies are provided in panel B. Results were compared using a Student's t-test (*p < 0.05 vs %FLAG detected by cTnI Ab). Western analysis of p-S150 is provided for FLAG-tagged and non-tagged phospho-mimetic cTnI in panel C. The relative change in p-S150 in myocytes expressing individual (e.g. S43D, S45D, S43/45D) or combined cTnI (SDTD, S4D) phospho- mimetics are compared in a representative Western blot in theFig. 2*.*
Data source location	*Ann Arbor, MI*
Data accessibility	*Data is in article.*

**Value of the data**

• The distribution of p-S23/24 and p-S150 between FLAG and endogenous cTnI is compared to the overall cTnI distribution in myocytes expressing FLAG-tagged or non-tagged cTnI with phospho-mimetic substitutions (Fig. 1; panels A and B).
• In myocytes expressing cTnIS4DFLAG, the p-S23/24 antibody does not recognize cTnIS4DFLAG (A, right panel) and thus, the distribution of p-S23/24 is not quantitatively analyzed for cTnIS4D.
• Secondary p-S150 in myocytes expressing cTnIS4D is compared to myocytes expressing cTnI, cTnISDTD, S43D and/or S45D (+FLAG; panel C and Fig. 2).

## 1 Data

**Fig. 1:**
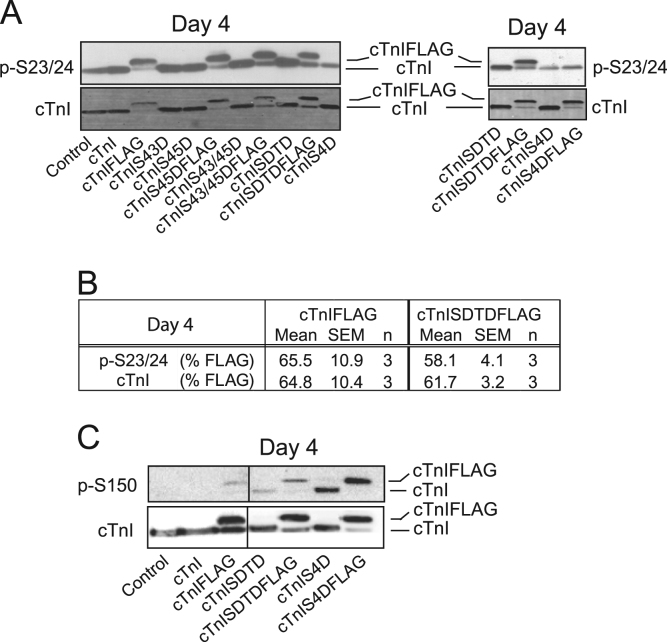
Representative blots (**A,C**) and table (**B**) showing phosphorylation in myocytes expressing FLAG-tagged or non-tagged cTnI with phospho-mimetic substitutions. **A.** Both panels show representative Western analysis of cTnI S23/24 phosphorylation (p-S23/24; upper panel) and cTnI (lower panel) in myocytes expressing cTnI with different phospho-mimetic substitutions. The left panel includes representative p-S23/24 for myocytes expressing cTnISDTD with and without FLAG on day 4 but lacks a lane with cTnIS4DFLAG. Thus, the absence of p-S23/24 detection is shown in myocytes expressing cTnI-SDTD versus -S4D with and without FLAG in the right panel. Our earlier work also included a quantitative analysis showing there were no significant changes in the phosphorylation and cTnI distribution for endogenous and FLAG tagged cTnI (Ref. [1]). This observation is verified for cTnISDTDFLAG in the panel **B** table. Values are expressed as the relative percentage of the FLAG/total cTnI ratio for p-S23/24 and for cTnI (e.g. MAB1691 Ab) in myocytes expressing cTnIFLAG and cTnISDTDFLAG. **C.** A representative Western blot also shows that the distribution of p-S150 and cTnI in myocytes expressing the FLAG-tagged cTnI phospho-mimetics. The line in the blot indicates a separation between cTnIFLAG and cTnISDTD on the same blot.

**Fig. 2:**
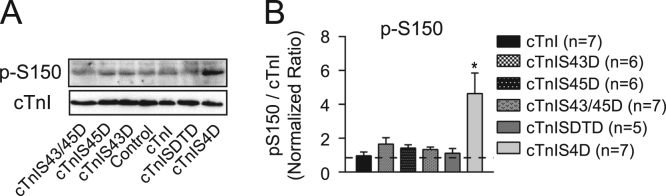
**A.** Representative Western analysis of S150 phosphorylation (p-S150) in cTnI for myocytes expressing cTnI with phospho-mimetic S43 and/or S45 alone or in combination with S23/24 (S4D) or T144 (SDTD) 4 days after gene transfer. **B.** Quantitative analysis of p-S150/cTnI ratio in the same groups shown in A. Results are calculated using the control ratio, which is set to 1.0 (dotted line) and compared to cTnI using a one-way ANOVA and post-hoc Tukey's test (*p < 0.05 vs cTnI).

Western blots are presented which detect secondary phosphorylation at S23/24 and S150 in myocytes expressing cTnI with phospho-mimetic substitutions at PKC-targeted residues. The S23/24 phosphorylation is quantitated in the panel B of the Fig. 1. The phosphorylated S150 data includes a comparison of myocytes expressing cTnI, cTnISDTD and cTnIS4D with and without a FLAG tag in panel C of the Fig. 1. A Fig. 2 compares pS150 in myocytes expressing non-tagged versions of these constructs and includes cTnI with individual substitutions at S43 and S45 evaluated in an earlier publication [1].

## 2 Experimental design, materials and methods

### 2.1 Site-directed mutagenesis and construction of adenoviral vectors for gene transfer

The cTnISDTD and cTnIS4D were produced by sequentially replacing cTnI-S43/45 followed by -T144 or -S23/24, respectively with negatively charged D in full-length, wild type cTnI cDNA (rat) via site-directed mutagenesis (QuikChange, Agilent Tech, Inc., Ref. [2]). Both FLAG-tagged and non-tagged versions of cTnISDTD and cTnIS4D were prepared in pGEM3Z [2], and then individually subcloned into a pDC315 shuttle vector. Recombinant adenovirus was produced by homologous recombination of each shuttle vector with pBHGLoxΔE1,3Cre in HEK293 cells [2], [3]. The mutagenesis primers for cTnIS23/24D were (mutations underlined) 5′-CTGCTCCTGTCCGACGTCGCGATGATGCCAACTACCGAGCCTATG-3′ (sense) and 5′-ATAGGCTCGGTAGTTGGCATCATCGCGACGTCGGACAGGAGCAG-3′ (anti-sense), and for cTnIT144D were 5′-GTGGCAAGTTTAAGCGGCCAGATCTCCGAAGAGTGAGAATC-3′ (sense) and 5′-GATTCTCACTCTTCGGAGATCTGGCCGCTTAAACTTGCCAC-3′ (anti-sense). Virus containing wild type cTnI (±FLAG), cTnI-S43D, -S45D (+FLAG), or -S43/45D (± FLAG) were prepared as described earlier [2].

### 2.2 Cardiac myocyte isolation and culture

Animal work was carried out according to handling protocols and procedures developed by the University Committee for the Use and Care of Animals (UCUCA) at the University of Michigan. Isolated Ca^2+^-tolerant adult rat myocytes were re-suspended in DMEM containing 5% fetal bovine serum (FBS), penicillin (50 U/ml) and streptomycin (50 μg/ml; P/S), and plated on laminin-coated coverslips at 37 °C for 2 h, as described earlier [2], [4]. Recombinant adenovirus diluted in serum-free M199 + P/S was added to myocytes at 37 °C and after one hour an 2 mL M199 + P/S aliquot was added to cells [4]. Media was changed 24 h after gene transfer and then every other day.

### 2.3 Expression analysis by Western blot

Prior to Western detection, cells were scraped into ice-cold sample buffer, and proteins were separated on 12% SDS polyacrylamide gels, electrophoretically transferred to PVDF membranes, and probed with primary and secondary antibody (Ab) pairs [2]. Protein phosphorylation at cTnI S23/24 (p-S23/24) and S150 (p-S150) and total cTnI expression were monitored by Western blot analysis 4 days after gene transfer [1], [2]. Primary antibodies (Abs) included TnI (MAB1691; Millipore), plus phospho-specific cTnI Abs for S23/24 (p-S23/24; Cell Signaling Technology) and S150 (p-S150; Ref. [5]). Each primary Ab-stained blot was then incubated with appropriate goat anti-mouse (GAM) or goat anti-rabbit (GAR) horseradish-peroxidase (HRP)-conjugated secondary Ab followed by enhanced chemilumenscence (ECL), which was detected with a ChemiDoc MP Imager (BioRad). A portion of each gel and blot was silver- and Sypro-stained, respectively. Quantitative analysis of Western blots, Sypro-stained blots, and silver (Ag)-stained gels was performed using Quantity One® software [1].
